# Supplementary material for: A large-scale population study of early life factors influencing left-handedness
Source: Sci Rep. 2019 Jan 24;9:584. doi: 10.1038/s41598-018-37423-8 (PMC6345846; doi:10.1038/s41598-018-37423-8)
Supplement: Supplementary file 1 — Supplementary information [file 41598_2018_37423_MOESM1_ESM.docx]

A large-scale population study of early life factors influencing left-handedness

Carolien G.F. de Kovel^a^, Amaia Carrión-Castillo^a^, Clyde Francks^a,b,^*

^a^ Department of Language and Genetics, Max Planck Institute for Psycholinguistics, Nijmegen, the Netherlands

^b^ Donders Institute for Brain, Cognition and Behaviour, Radboud University Nijmegen, Nijmegen, The Netherlands

Supplementary information

## Supplemental tables, figures, and exploratory analyses

### Supplemental Tables

Supp. Table 1. Univariable analysis of early life variables and handedness, male participants

Supp. Table 2. Univariable analysis of early life variables and handedness, female participants

Supp. Table 3 Multivariable model for right-handedness, male participants

Supp. Table 4 Multivariable model for right-handedness, female participants

Supp. Table 5. Heritability estimates of handedness and covariates of interest

Supp. Table 6. Genetic correlation of handedness with a second trait

### Supplemental Figures

Supp. Figure 1 Male and female participants by year of birth

Supp. Figure 2 Left-handedness vs year of birth

Supp. Figure 3 Left-handedness vs birthweight

Supp. Figure 4 Left-handedness vs month of birth

Supp. Figure 5 Birthweight vs year of birth

Supp. Figure 6 Birthweight vs month of birth

Supp. Figure 7. Power analysis for genetic correlation

Supp. Figure 8. Genetic correlations between variables

### Supplemental Analyses

Additional models for season

Additional analysis of low birthweight and multiple birth

# Tables

Supp. Table S1. Univariable analysis of categorical variables for handedness in males only

| **MALES Categorical** | **N** | **p-value** | **Frequency of left-handedness** | **Notes** | **OR right-handed (95% C.I.)** |
| --- | --- | --- | --- | --- | --- |
| **part of multiple birth** | 189,180 | 0.007 | non-twin = 10.6%, twin=11.9% | 2.2% of participants are from multiple birth | 0.88 (0.80-0.96) |
| **maternal smoking** | 164,876 | 0.096 | non-smoking=10.5%; smoking=10.8% | 30% of mothers smoked around pregnancy | 0.97 (0.94-1.01) |
| **breastfeeding** | 151,613 | 6.3E-23 | breastfed=10.2%, not breastfed=12.0% | 76% of participants were breastfed | 1.20 (1.16-1.25) |
| **country of origin** | 215,422 | 5.1E-58 |  | Lowest frequency non-right-handers born outside UK, highest in England |  |
| England | 148,688 |  | 11.3% |  |  |
| Northern Ireland | 1,341 |  | 10.1% |  | 1.13 (0.95-1.36) |
| Scotland | 15,532 |  | 9.3% |  | 1.23 (1.16-1.30) |
| Wales | 8,446 |  | 8.7% |  | 1.34 (1.24-1.45) |
| Republic of Ireland | 2,032 |  | 8.2% |  | 1.42 (1.21-1.66) |
| Elsewhere | 16,161 |  | 7.7% |  | 1.51 (1.42-1.60) |
| **MALES Continuous** | **N** | **p-value** | **Note** | **Effect size on right-handed** | |
| **year of birth** | 192,569 | 6.5E-14 | Increase left-handers ~ 0.7 percentage-point per decade | Effect = -0.007 yr^-1^ | |
| **year squared** |  | 0.058 |  |  | |
| **birthweight** | 89,924 | 0.001 | Left-handers are ~24g lighter on average | Effect = 0.052 kg^-1^ | |
| **cosine(month)** | 192,569 | 0.388 | See Figure S4 | Effect = 0.006 | |

Supp. Table S2. Univariable analysis for handedness in females only

| FEMALES Categorical | **N** | **p-value** | **Frequency of left-handedness** | **Notes** | **OR right-handed (95% C.I.)** |
| --- | --- | --- | --- | --- | --- |
| part of multiple birth | 225,380 | 5.9E-07 | non-twin = 8.5%, twin=10.5% | 2.3% of participants are from multiple birth | 0.79 (0.72-0.87) |
| maternal smoking | 198,990 | 0.914 | non-smoking=8.5%; smoking=8.6% | 30% of mothers smoked around pregnancy | 1.0 (0.96-1.03) |
| breastfeeding | 185,231 | 1.1E-13 | breastfed=8.3%, not breastfed=9.3% | 70% of participants were breastfed | 1.14 (1.10-1.18) |
| country of origin | 228,739 | 8.7E-88 |  | Lowest frequency non-right-handers born outside UK, highest in England |  |
| England | 196,351 |  | 9.3% |  |  |
| Northern Ireland | 1,558 |  | 7.8% |  | 1.21 (1.01-1.47) |
| Scotland | 18,892 |  | 7.1% |  | 1.33 (1.26-1.41) |
| Wales | 9,924 |  | 6.3% |  | 1.52 (1.39-1.65) |
| Republic of Ireland | 2,769 |  | 6.6% |  | 1.44 (1.24-1.69) |
| Elsewhere | 21,997 |  | 6.0% |  | 1.59 (1.50-1.69) |

| **FEMALES, continuous** | **N** | **p-value** | **Note** | **Effect size on right-handed** |
| --- | --- | --- | --- | --- |
| **year of birth** | 229,098 | 7.3E-23 | Increase left-handed ~ 0.8 percentage-point per decade | Effect = -0.009 yr^-1^ |
| **year squared** |  | 0.014 | - | - |
| **birthweight** | 141,231 | 3.1E-06 | Left-handers are ~28g lighter on average | Effect = 0.068 kg^-1^ |
| **cosine(month)** | 255,401 | 0.0012 | See Figure S4 | Effect = 0.034 |

Supp. Table S3. Multivariable logistic model for right-handedness, male participants

| **MALES Variable** | **Estimate** | **S.E** | **z** | **p-value** |  | **OR right-handed (95% C.I.)** |
| --- | --- | --- | --- | --- | --- | --- |
| (Intercept) | 14.207 | 2.71 | 5.241 | 8.0E-08 |  |  |
| **Categorical** |  |  |  |  |  |  |
| **multiple birth (Yes)** | -0.15 | 0.07 | -2.13 | 0.033 |  | 0.86 (0.76-0.99) |
| **breastfed (Yes)** | 0.15 | 0.02 | 5.87 | 4.3E-09 |  | 1.16 (1.10-1.22) |
| **country-Ireland** | 0.27 | 0.16 | 1.72 | 0.085 |  | 1.31 (0.98-1.81) |
| **country-NI** | 0.09 | 0.14 | 0. 61 | 0.544 |  | 1.09 (0.83-1.47) |
| **country-Scotland** | 0.19 | 0.04 | 4.24 | 2.3E-05 |  | 1.20 (1.11-1.31) |
| **country-Wales** | 0.35 | 0.06 | 6.02 | 1.8E-09 |  | 1.43 (1.27-1.60) |
| **country-Elsewhere** | 0.30 | 0.05 | 5.560 | 2.7E-08 |  | 1.35 (1.22-1.51) |
| **Continuous** |  |  |  |  |  |  |
| **year** | -0.006 | 0.001 | -4.58 | 4.7E-06 |  |  |
| **birthweight** | 0.040 | 0.017 | 2.31 | 0.021 |  |  |
| **Model information** |  |  |  |  |  |  |
| McFadden pseudo R^2^ | 0.003 |  |  |  |  |  |
| Log likelihood vs null | p=1.2E-31 | | |  |  |  |
| Hosmer Lemeshow test | p=0.80 | | |  |  |  |
| N | 83,506 | | |  |  |  |

Supp. Table S4. Multivariable logistic model for right-handedness, female participants

| **FEMALES Variable** | **Estimate** | **S.E** | **z** | **p-value** |  | **OR right-handed (95% C.I.)** |
| --- | --- | --- | --- | --- | --- | --- |
| (Intercept) | 2.058 | 0.053 | 38.60 | 0.00E+00 |  |  |
| **Categorical** |  |  |  |  |  |  |
| **multiple birth (Yes)** | -0.137 | 0.057 | -2.426 | 0.015 |  | 0.87 (0.78-0.98) |
| **breastfed (Yes)** | 0.078 | 0.021 | 3.737 | 1.9E-04 |  | 1.08 (1.04-1.13) |
| **country-Ireland** | 0.216 | 0.120 | 1.349 | 0.072 |  | 1.24 (0.99-1.58) |
| **country-NI** | 0.263 | 0.136 | 1.797 | 0.052 |  | 1.30 (1.01-1.72) |
| **country-Scotland** | 0.296 | 0.038 | 7.790 | 6.7E-05 |  | 1.35 (1.25-1.45) |
| **country-Wales** | 0.461 | 0.054 | 8.582 | 4.7E-19 |  | 1.59 (1.43-1.73) |
| **country-Elsewhere** | 0.333 | 0.043 | 7.825 | 5.1E-15 |  | 1.40 (1.29-1.52) |
| **Continuous** |  |  |  |  |  |  |
| **year** | -0.007 | 0.001 | -5.918 | 3.3E-09 |  |  |
| **year^2 (scaled)** | 4.794 | 3.55 | 1. 35 | 0.177 |  |  |
| **birthweight** | 0.051 | 0.016 | 3.20 | 0.0014 |  |  |
| **month.cos** | 0.038 | 0.014 | 2.844 | 0.0045 |  |  |
| **Model information** |  |  |  |  |  |  |
| McFadden pseudo R^2^ | 0.003 |  |  |  |  |  |
| Log likelihood vs null | p=7.3E-54 | | |  |  |  |
| Hosmer Lemeshow test | p=0.454 | | |  |  |  |
| N | 136,488 | | |  |  |  |

Supp. Table S5. Heritability estimates of handedness, birthweight and being breastfed

| **Trait** | **N** | **N_cases_^a^** | **Prevalence^b^** | **SNP-h^2^ (SE)** | **p-value** |
| --- | --- | --- | --- | --- | --- |
| **handedness (left)** | 330,474 | 32,367 | 0.098 | 0.0435 (0.0016)^c^ | 1.59e-19 |
| **birthweight** | 191,648 | - | - | 0.1547 (0.0034) | 0 |
| **breastfed (yes)** | 254,620 | 180,724 | 0.71 | 0.0594 (0.0024)^c^ | 6.9e-156 |

^a^N_cases_: number of ‘cases’ for binary traits. ^b^Prevalence: frequency of cases in the analysed sample. ^c^Heritability estimate in the liability scale. P-values are calculated as pr(X2=(estimate/se)^2^,df=1).

Supp. Table S6. Genetic correlation (ρ) of handedness with birthweight and being breastfed. P-values are calculated as pr(X2=(estimate/se)^2^,df=1).

| **Trait** | **ρ (SE)** | **p-value** | **N** |
| --- | --- | --- | --- |
| **birthweight** | 0.0212 (0.042) | 0.615 | 188,529 |
| **breastfed** | 0.0165 (0.071) | 0.816 | 250,498 |

# Supplemental figures


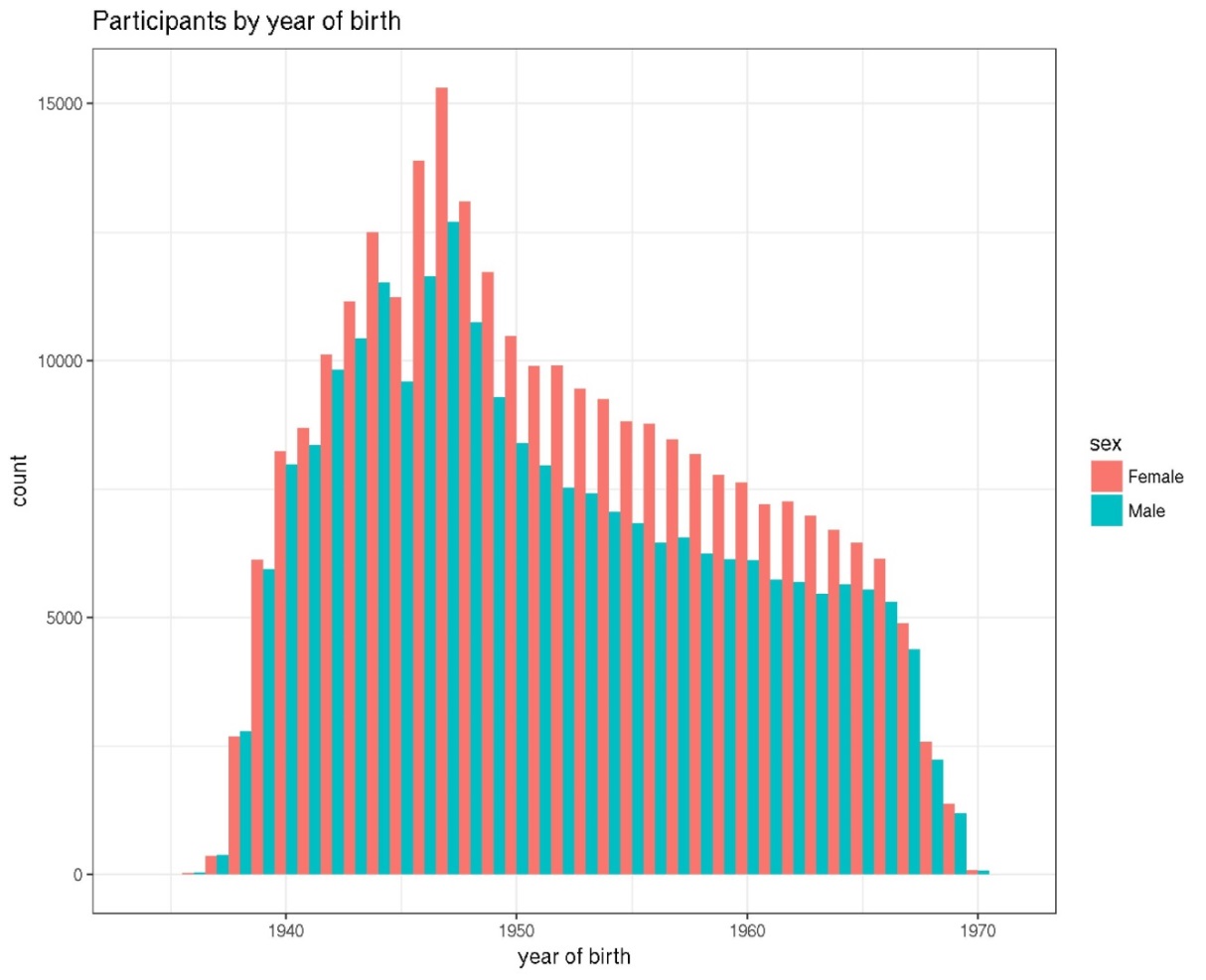


Supp. Figure 1 Male (green) and female (orange) participants in the UK biobank cohort by year of birth


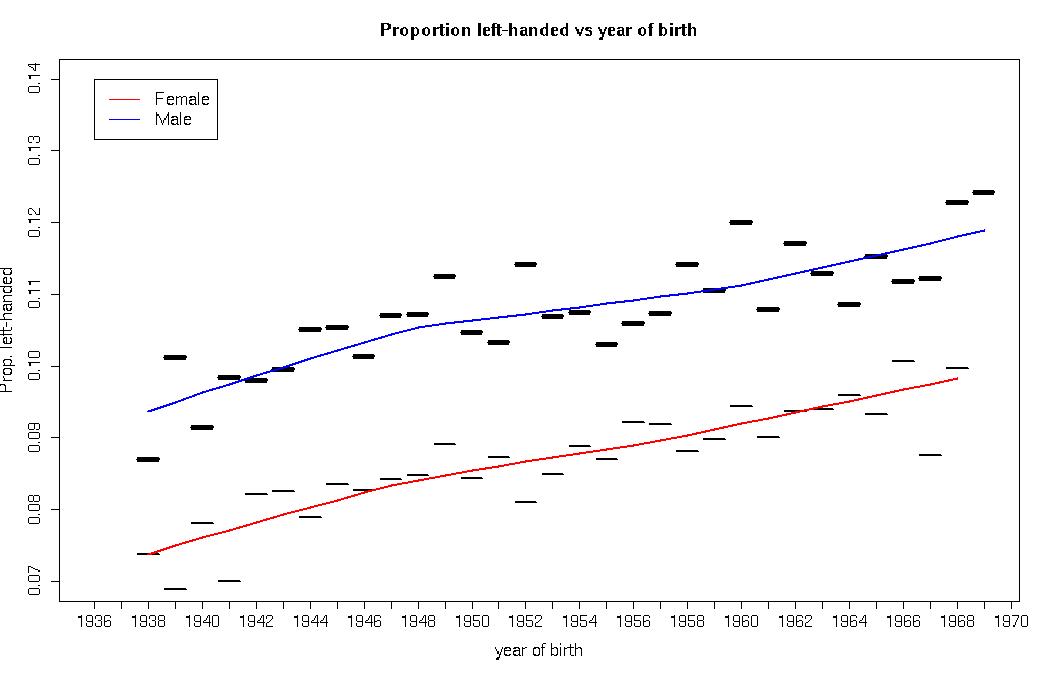


Supp. Figure 2 Frequency of left-handers by year of birth for males (fat, with blue lowess line)) and females (thin, with red lowess (=locally-weighted polynomial regression) line). Years with < 100 participants excluded. Note Y-axis does not start at zero.


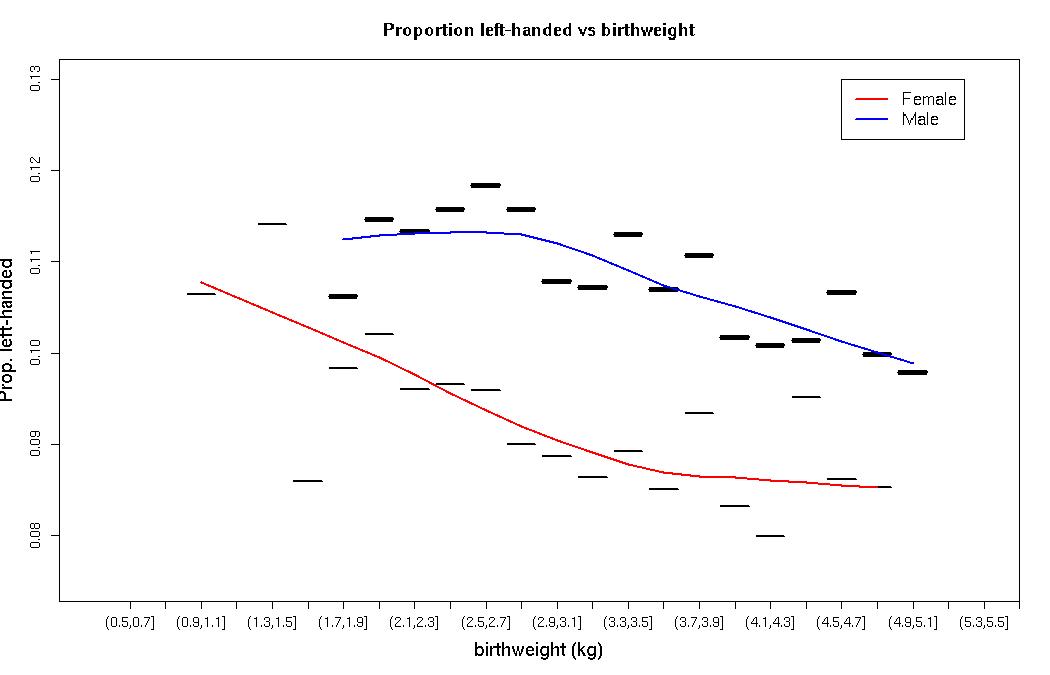
Supp. Figure 3 Proportion left-handers by birthweight bins, for males (fat symbols, with blue lowess line)) and females (thin, with red lowess line). Bins of 200 g; bins with < 50 samples discarded. Note Y-axis does not start at zero.


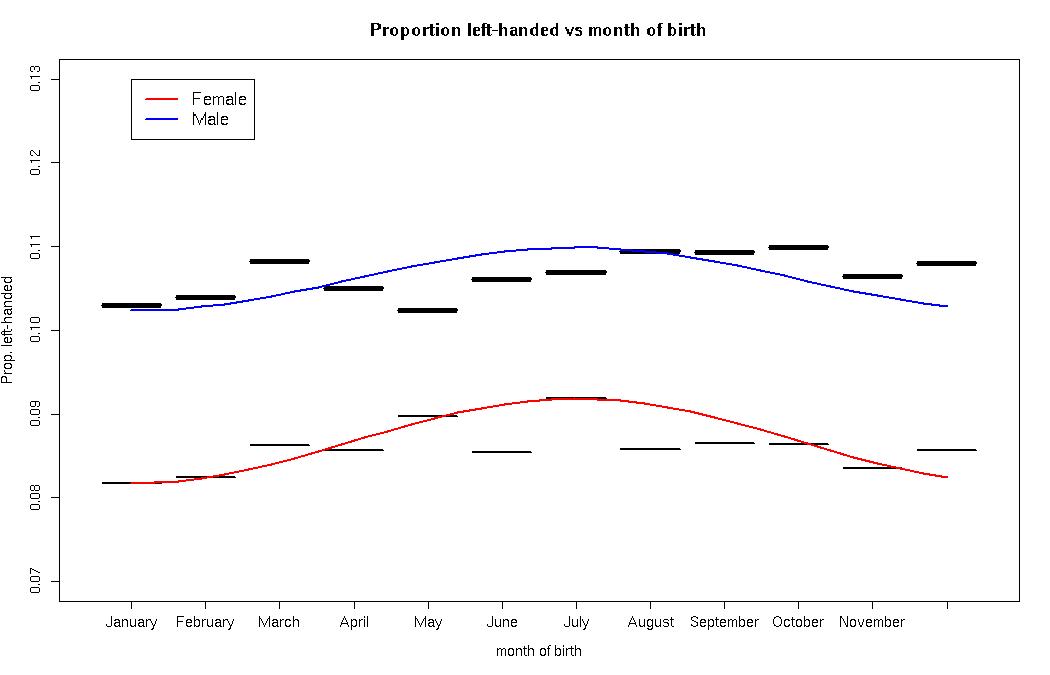


Supp. Figure 4 Proportion of left-handers by month of birth for males (fat, blue cosine) and females (thin, red cosine) with overlay of the primary cosine of month model scaled to the data. Note Y-axis does not start at zero.


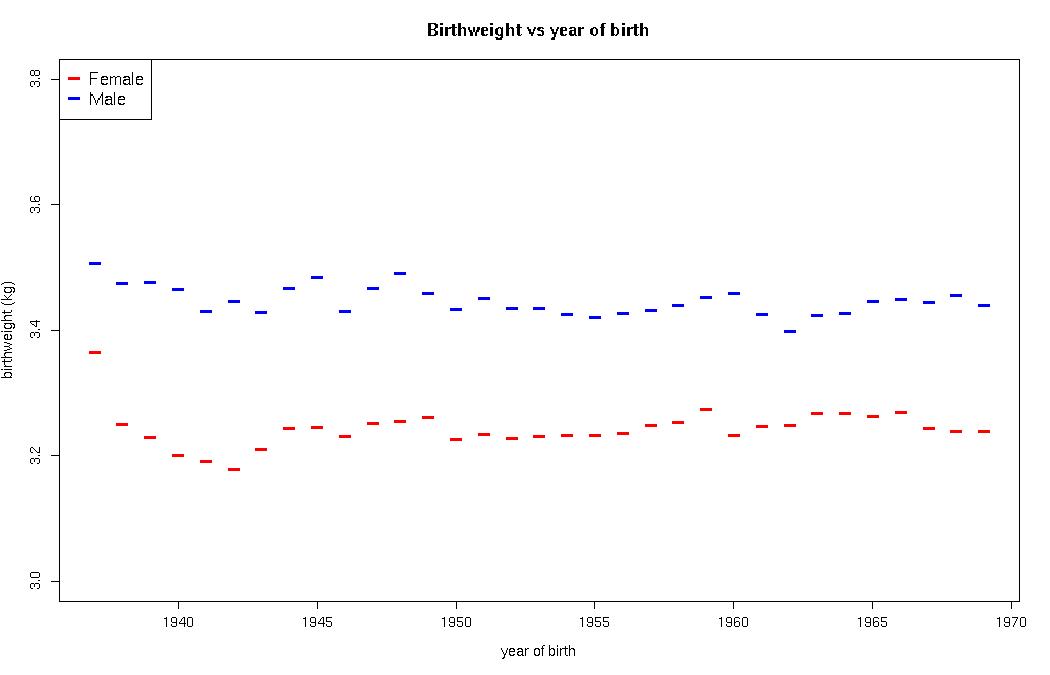


Supp. Figure 5 Average birthweight (kg) vs year of birth for males (blue) and females (red). Note Y-axis does not start at zero. In order not to obscure the general pattern, no error bars are presented.


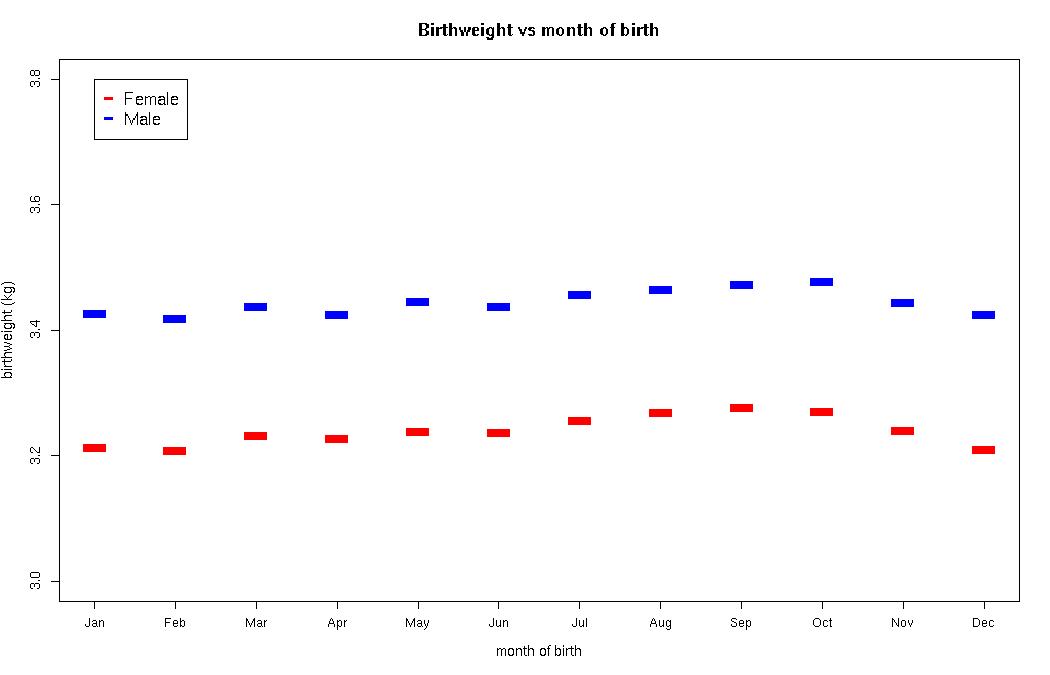


Supp. Figure 6 Average birthweight (kg) vs month of birth. Note Y-axis does not start at zero. In order not to obscure the general trend, no error bars are presented.


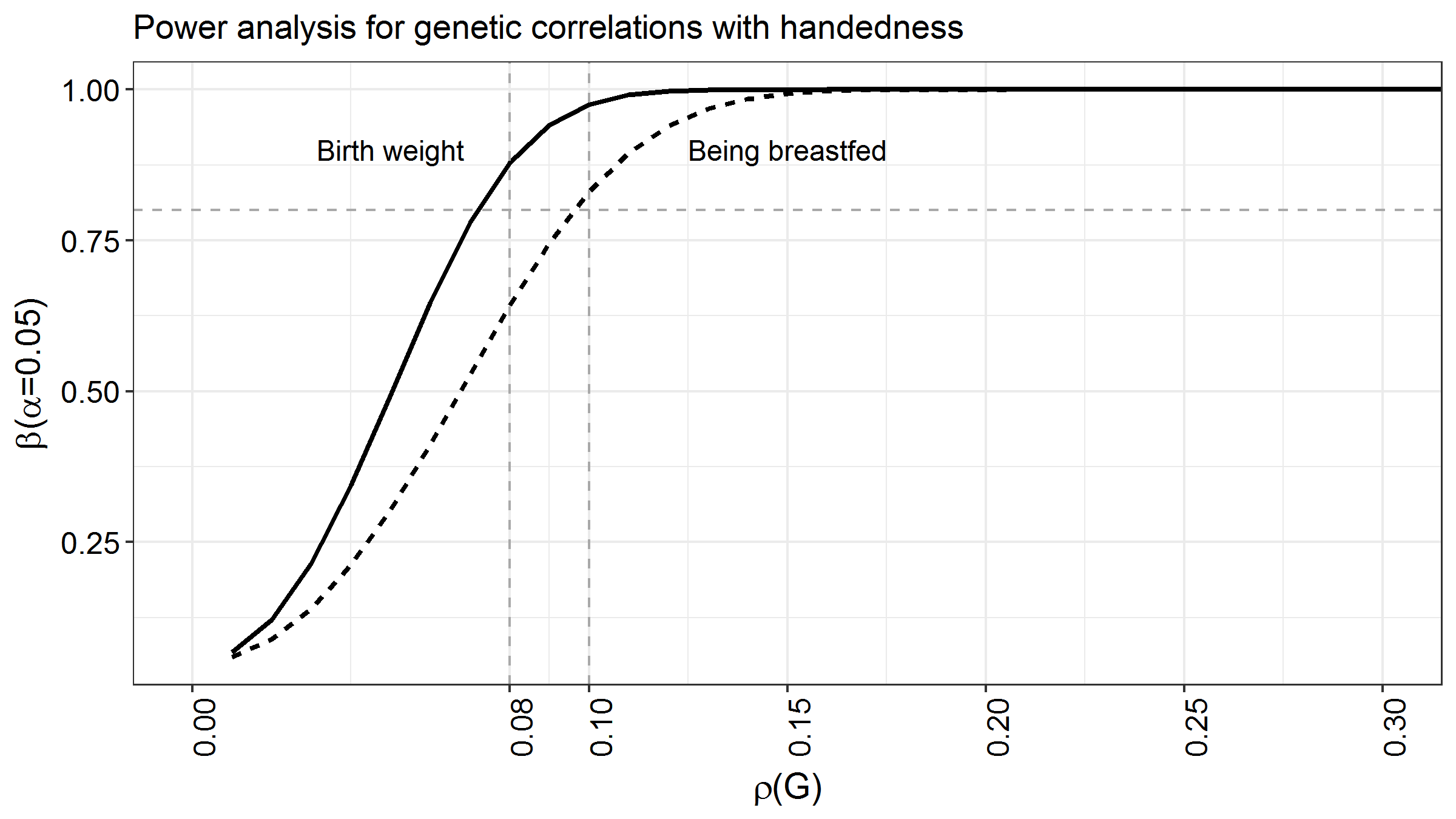


Supp. Figure 7 . Power analysis for genetic correlation (ρ) between handedness and either birthweight or being breastfed. Sample size, heritability and prevalence are modelled as indicated in Supp. Table 5. The x axis indicates genetic correlation, the y axis statistical power. Power is lower for the binary trait of being breastfed versus the continuous measure birthweight.

# Supplemental analyses

## Additional models for season

In our primary analysis we included a quantitative predictor variable which was the cosine of month of birth (Methods), which assumed that any effect of season on hand preference would follow seasonal extremes of daylight and temperature in the UK, i.e. with its maximum and minimum in January and July respectively (the direction is arbitrary). However, other seasonal variables with minima and maxima at other times of year may be more important, or there may be sensitive periods at earlier moments during pregnancy. We therefore explored this issue further by designing a family of six cosine functions, each shifted by one month with respect to the next (a further six models would then be negative specifications of one of the first six, and would therefore give identical results but in reverse direction). All models were of the same shape as in the primary analysis:

Cos(2π(*t*_i_−m)/12)

With t_i_ as the month of the individual’s birth, and m varying from 1 to 6 (Supp. Figure 8).


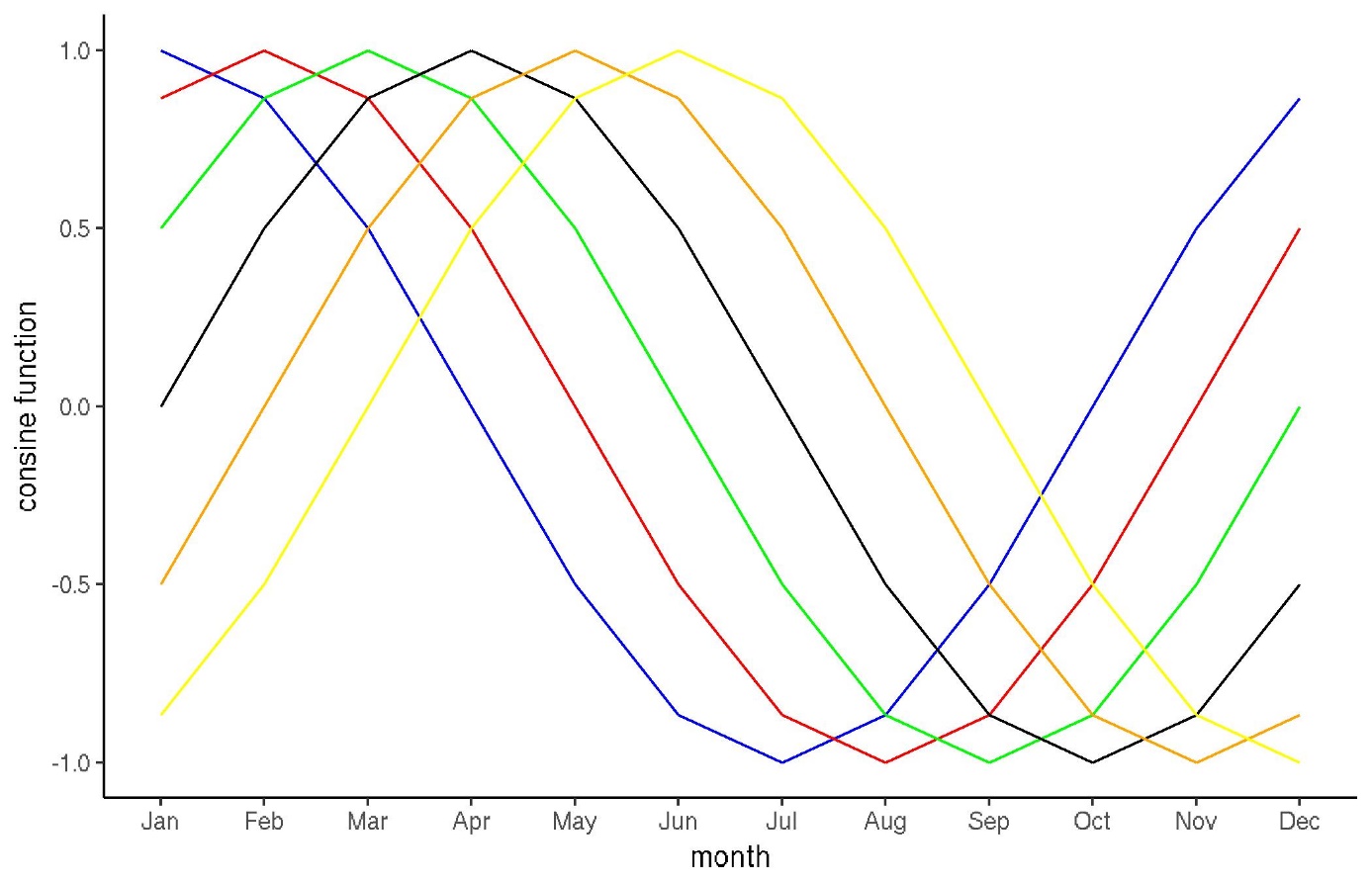


Supp. Figure 8 Family of cosine-based models to capture seasonal effects.

As our main analysis had suggested a more significant seasonal effect in females, we ran the analysis within the separate sexes, as well as combined (Supp. Tables 7, 8, 9). This exploratory analysis was done using logistic regression on a univariable basis, i.e. with hand preference as a function only of the cosine variable (see Methods).

Supp. Table 7 Seasonal effects for both sexes (negative effects mean an increase in left-handedness among those born in that month)

| Maximum | Effect | T |  | p | Colour in Supp. Fig. 8 |
| --- | --- | --- | --- | --- | --- |
| JAN | 0.022 | 2.91 |  | 0.0036 | Blue |
| FEB | 0.023 | 3.10 |  | 0.0020 | Red |
| MAR | 0.018 | 2.45 |  | 0.014 | Green |
| APR | 0.009 | 1.15 |  | 0.25 | Black |
| MAY | -0.003 | -0.458 |  | 0.65 | Orange |
| JUN | -0.015 | -1.974 |  | 0.051 | Yellow |

Supp. Table 8 Seasonal effects for males (negative effects mean an increase in left-handedness among those born in that month)

| Maximum | Effect | T |  | p | Colour in Supp. Fig. 8 |
| --- | --- | --- | --- | --- | --- |
| JAN | 0.009 | 0.86 |  | 0.39 | Blue |
| FEB | 0.019 | 1.83 |  | 0.068 | Red |
| MAR | 0.024 | 2.30 |  | 0.021 | Green |
| APR | 0.023 | 2.16 |  | 0.031 | Black |
| MAY | 0.015 | 1.44 |  | 0.15 | Orange |
| JUN | 0.003 | 0.33 |  | 0.74 | Yellow |

Supp. Table 9 Seasonal effects for females (negative effects mean an increase in left-handedness among those born in that month)

| Maximum | Effect | T |  | p | Colour in Supp. Fig. 8 |
| --- | --- | --- | --- | --- | --- |
| JAN | 0.034 | 3.24 |  | 0.0012 | Blue |
| FEB | 0.027 | 2.52 |  | 0.012 | Red |
| MAR | 0.012 | 1.13 |  | 0.26 | Green |
| APR | -0.006 | -0.56 |  | 0.58 | Black |
| MAY | -0.022 | -2.11 |  | 0.035 | Orange |
| JUN | -0.033 | -3.09 |  | 0.0020 | yellow |

As can be seen (Supp. Tables 7, 8, 9), for both sexes combined, the strongest effect is with a cosine function with the minimum rate of left-handedness among those born in February, and maximum in August, similar to that used in our primary analysis (minimum January, maximum July)(Supp. Table 7, see also Supp. Fig. 4). This indicates that left-handedness is most common in babies born in summer. Splitting by sex, the effect in females was again largest for a cycle indicating the highest rate of left-hand preference among July births, minimum January, as used in our primary analysis. The effect in males was less significant and offset to a cycle with its maximum left-handedness rate among those born in September, although only nominally significant, and not visually apparent in the data (Supp. Fig. 4).

## Additional analysis of low birthweight and multiple birth

Earlier studies (by the same group)^1,2^ had suggested that the association between birthweight and hand preference may be more pronounced at low birthweights, an observation which was investigated in depth in triplets. Note that the UK Biobank variable ‘Part of a multiple birth’ makes no distinction between twins, triplets, quadruplets etc., although the large majority are expected to be twins. We analysed the individuals in the UK Biobank dataset with birthweight < 2kg as a distinct subset (N=7,498). Within this subset, the range in birthweight was 0.45 – 1.98kg, and there were 786 left-handers and 1637 from multiple births (199 subjects were both left-handed and from multiple births). Unsurprisingly, in this subset, the proportion of multiple births was considerably higher than in the total UK Biobank dataset (21.8% of subjects vs 2.2% in the total dataset). Again within this subset, 10% of the singletons were left-handed, while 12.2% of those from multiple births were left-handed (chi-square test, p=0.014). As this was a subset selected from the tail of the birthweight distribution, birthweight was not normally distributed within it (Supp. Fig. 9), and we therefore used the non-parametric Kruskal-Wallis test to compare birthweight between hand preference groups, and between singletons versus those from multiple births.

Those from multiple births still had lower mean birthweights (1.536 kg) than singletons (1.581 kg)(Kruskal-Wallis test p=1.5E-07, Supp. Figure 10).Those from multiple births had a mean birthweight of 1.547 kg when left-handed and 1.537kg when right-handed (though note that the mean is not a very good descriptor in this distribution). This difference was not significant, p=0.71. Among the singletons, the left-handers had a mean birthweight of 1.553kg compared with right-handers 1.585 kg (p=0.06). Combining single and multiple births, left-handers had a mean birthweight of 1.548 kg versus right-handers 1.573kg (p=0.09). These group differences by handedness are similar in magnitude (see Supp. Fig. 10) to the overall UK Biobank dataset (See Table 3 in the main text). The finding of no increased association between birthweight and hand preference in this subset is also in agreement with the roughly linear trend in the total dataset, visible in Supp. Figure 3.


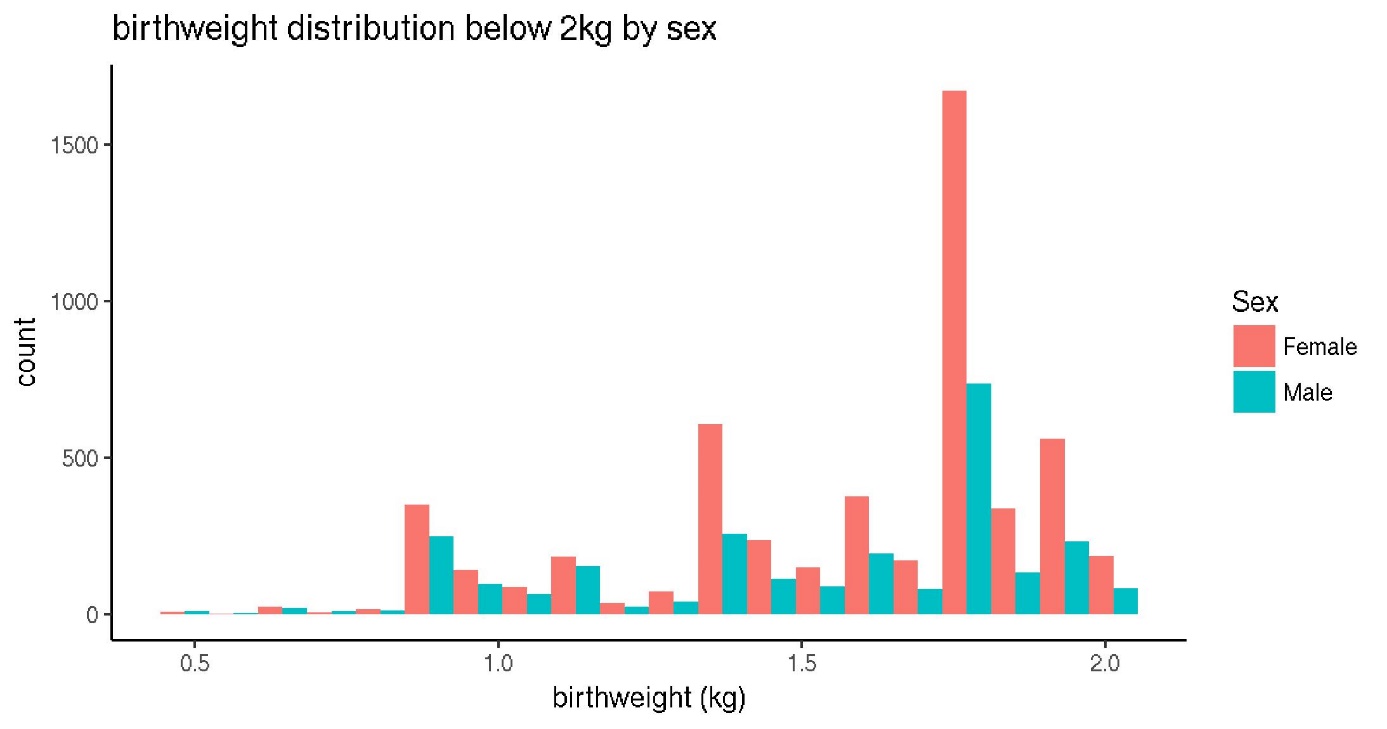


Supp. Figure 9. Birthweight distribution in those born with birthweights less than 2kg (shown here separately by sex).


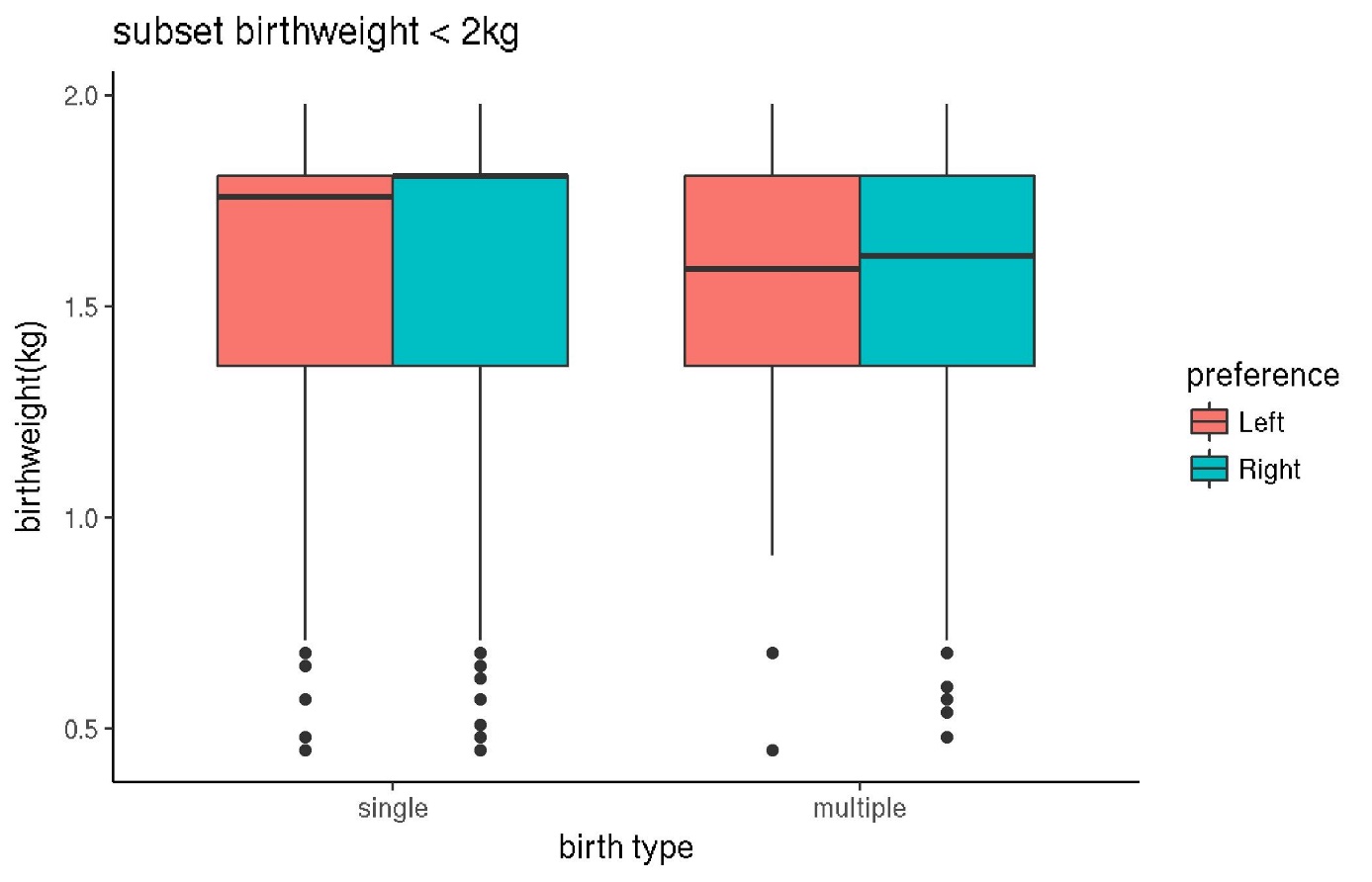


Supp. Figure 10. Birthweight by hand preference and multiple birth in the subset with birthweight < 2 kg. Coloured box entends from 1^st^ to 3^rd^ quartile, bold horizontal line is median.

Finally, we ran the same multivariable model as the main analysis, but only within the individuals from multiple births (then excluding multiple birth as a predictor variable)(N(right-handed)=5064, N(left-handed)=649). Clearly the statistical power of this analysis was limited compared to the much larger main analysis. The only detectable effects on hand preference in this multivariable model were sex (OR=0.78, p=0.003), and a weak effect of being born outside the UK (OR=1.6, p=0.038). The p-value for the likelihood ratio of the model was p=0.014.

# Supplemental references

1 Heikkila, K. *et al.* Triplets, birthweight, and handedness. *Proc Natl Acad Sci U S A* **115**, 6076-6081, doi:10.1073/pnas.1719567115 (2018).

2 Heikkila, K. *et al.* Higher Prevalence of Left-Handedness in Twins? Not After Controlling Birth Time Confounders. *Twin Res Hum Genet* **18**, 526-532, doi:10.1017/thg.2015.53 (2015).
